# Positive impact of a patient-perspective video on engagement and learning in a kidney transplant seminar: a cluster-randomized study

**DOI:** 10.1007/s00345-026-06680-y

**Published:** 2026-08-17

**Authors:** Marcus Derigs, Jonas Beckmann, Fabian Kormann, Fabian Derigs, Christer Groeben, Luka Flegar, Philipp Reimold, Rainer Koch, Johannes Huber

**Affiliations:** 1 Marburg University, Department of Urology, Marburg, Hesse Germany; 2 Department of Urologic Sciences, The Stone Centre at Vancouver General Hospital, University of British Columbia, Vancouver, BC Canada; 3 Department of Urology, University Hospital Heidelberg, Heidelberg, Baden- Wuerttemberg, Germany

**Keywords:** Kidney transplantation, Medical education, Multimedia learning, Student engagement, Emotional engagement

## Abstract

**Objectives:**

Patient narratives may enhance medical students’ attention and memory. We tested whether a brief patient-perspective video shown at the beginning of a kidney transplantation seminar was associated with improved immediate knowledge retention and emotional engagement.

**Methods:**

In a cluster-randomized design, 154 third-year medical students were assigned by session to a Video Group (*n* = 77) or Control Group (*n* = 77). The Video Group watched a 4-minute patient-perspective video on kidney transplantation before the standardized 60-minute lecture, whereas the Control Group received the lecture only. The primary outcome was post-seminar knowledge retention, assessed using a 10-item multiple-choice quiz. Secondary outcomes were self-reported emotional engagement, interest in the topic, seminar satisfaction, and perceived contribution of media use to understanding, each rated on a 5-point Likert scale.

**Results:**

The Video Group scored higher on the post-seminar quiz than the Control Group (mean ± standard deviation: 6.14 ± 1.79 vs. 4.95 ± 1.78; *p* < 0.001), with a medium effect size (Cohen’s *d* = 0.67). The Video Group also reported stronger emotional engagement with the seminar topic (*p* = 0.012). No significant differences were found between groups for the other evaluation items.

**Conclusion:**

A brief patient-perspective video shown before a kidney transplantation seminar was associated with higher immediate knowledge retention and greater self-reported emotional engagement. Short patient-perspective videos may be a low-cost, scalable adjunct to traditional teaching.

**Supplementary Information:**

The online version contains supplementary material available at 10.1007/s00345-026-06680-y.

## Introduction

Contemporary medical education faces mounting challenges as the volume of medical knowledge expands rapidly [1], while time for formal teaching and clinical exposure remains limited [2]. This imbalance underscores the need for efficient teaching methods.

One approach is to expose learners to patients’ lived experiences of illness, which can provide clinical relevance and emotional resonance. Traditionally delivered in lectures or at the bedside [3, 4], patient narratives have also been used in recorded formats. Widespread digital tools now make these narratives more accessible and easier to produce, supporting broader and more flexible use in medical school curricula [5, 6]. Video formats also offer advantages in availability, privacy, and scheduling efficiency. Although patient-perspective videos have shown promise, key questions remain about optimizing their educational impact, particularly regarding content, timing, and curricular integration.

These materials may be especially useful because they can foster emotional engagement and reflection [7]. Combined with evidence that moderate emotional arousal heightens attention and supports memory formation [8], this suggests that intentionally eliciting appropriate emotional arousal may be one way for patient-perspective videos to enhance learning.

Kidney transplantation is a suitable topic for evaluating such strategies, given its clinical importance, emotional salience, and underrepresentation in undergraduate curricula [9].

We introduced a 4-minute patient-perspective video at the start of a urology seminar on kidney transplantation and assessed its impact using a cluster-randomized design. The primary outcome was immediate knowledge retention, measured by a post-seminar multiple-choice quiz. Secondary outcomes were emotional engagement, interest, satisfaction, and perceived contribution of media use to understanding. We hypothesized that exposure to the video would increase engagement and improve quiz performance compared with a standard seminar without the video.

## Materials and methods

### Study setting and participants

This cluster-randomized study was conducted at the Faculty of Medicine at the University of Marburg (Philipps-Universität Marburg), Germany, as part of a compulsory third-year urology block. The 90-minute kidney transplantation seminar was the final session of the six-part block. Students had no prior coursework on kidney transplantation.

A total of 154 third-year medical students participated. They were divided into 12 seminar groups across six teaching dates within a nine-day period, with two concurrent sessions held on each date. Each seminar was led by one of three urologists, referred to as Lecturers A, B, and C. Seminar content and format were standardized, and instructor assignments were rotated so that each lecturer taught both Video and Control sessions.

## Randomization and intervention

Each pair of concurrent sessions was cluster-randomized to the Video or Control condition. Cluster randomization was chosen for feasibility and to match seminar logistics: two concurrent groups per date, with 12 groups total across nine days. Individual randomization was not practicable in this setting.

In the Video Group, students watched a 4-minute patient-perspective video at the start of the seminar (Supplementary Video S1). The video depicted a living-donor kidney transplantation and included the recipient, her mother as the living donor, and the treating surgeon (J.H.). It emphasized the human context of transplantation rather than detailed medical content. The video was professionally produced for a general audience by Hessischer Rundfunk (producer: Kim Horbach) under the medical supervision of J.H. Intervention fidelity was supported by using the same slide deck across sessions, a standardized lecture outline and timing for all three lecturers, and fixed video placement at the start of the class.

Control sessions did not include the video or any replacement activity. Both groups received the same 60-minute instructor-led lecture on kidney transplantation, followed by 10 min for questions and discussion. To minimize contamination between groups, students were asked not to discuss the seminar content or study procedures with peers until all sessions had been completed. As a small incentive, students received a chocolate bar after participation.

## Outcomes

The prespecified primary outcome was post-seminar quiz score, ranging from 0 to 10. Each question had five options and one correct answer. Items were mapped to predefined learning objectives, including indications, surgical technique, immunosuppression, complications, and legal/historical aspects, following principles of test blueprinting to support content coverage across the seminar [10]. All items were authored and independently reviewed by the three urologists to ensure coverage across the seminar’s content. The instrument was not psychometrically validated.

Secondary outcomes were assessed using an evaluation form including one yes/no item on overall enjoyment of the seminar and four 5-point Likert items assessing interest in the topic, emotional engagement, satisfaction with seminar content, and perceived contribution of media use to understanding (Supplementary File S1). The form was derived from the medical school’s course evaluation template. At each session’s end, students completed the quiz and evaluation anonymously on paper. Quizzes were ungraded, and no feedback was provided until study completion. One blank evaluation form from a Control session was excluded.

### Statistical analysis

Quiz scores were compared using an unpaired two-tailed *t*-test, and effect size was calculated as Cohen’s *d*. Two two-way ANOVAs assessed consistency across instructors and seminar dates using Type III sums of squares; non-significant interaction terms were removed. Likert-scale responses were compared using Cochran-Armitage trend tests. A two-sided *p*-value < 0.05 was considered significant. Analyses were performed using GraphPad Prism v10 and SAS v9.4.

## Results

### Participant characteristics

A total of 154 students participated across 12 sessions on six dates (Video Group, *n* = 77; Control Group, *n* = 77). Each instructor taught four sessions. The overall mean quiz score was 5.55 ± 1.88 out of 10, with slight variation by instructor, ranging from approximately 5 for Lecturer B to 6 for Lecturer A (Supplementary Table S1).

## Video introduction was associated with higher quiz scores

**Fig. 1:**
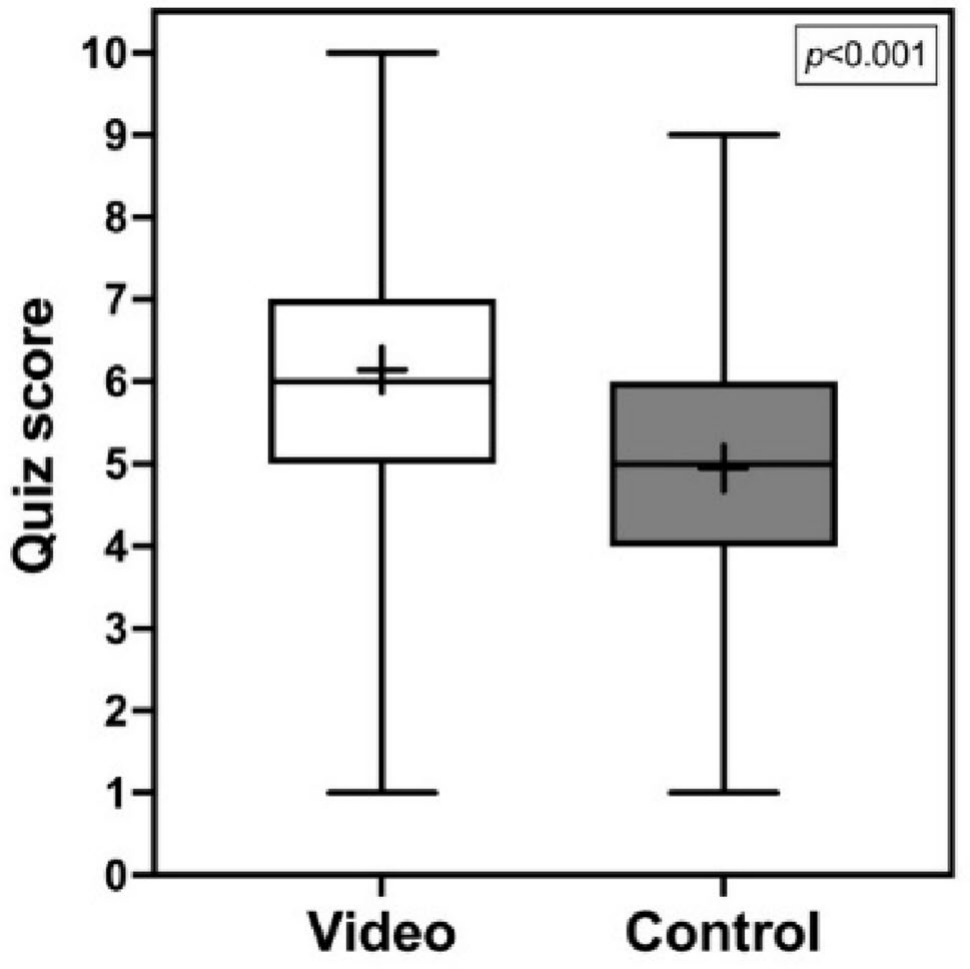
Effect of a patient-perspective video introduction on student quiz scores

Students who viewed the patient-perspective video achieved higher quiz scores than those who did not (6.14 ± 1.79 vs. 4.95 ± 1.78). This corresponded to a mean difference of 1.19 points on the 10-item quiz and was statistically significant (*p* < 0.001; Fig. 1). The standardized effect size was Cohen’s *d* = 0.67.

Third-year medical students who viewed a 4-minute patient-perspective video before a kidney transplantation seminar (Video Group, *n* = 77) scored higher on a 10-item multiple-choice quiz than those who did not view the video (Control Group, *n* = 77). Box plots show the median, interquartile range (25th–75th percentile), range, and the mean (+). The Video Group’s mean score was 6.14 ± 1.79, compared with 4.95 ± 1.78 in the Control Group (unpaired *t*-test, *p* < 0.001).

## Quiz score difference was consistent across lecturers and seminar dates

**Fig. 2:**
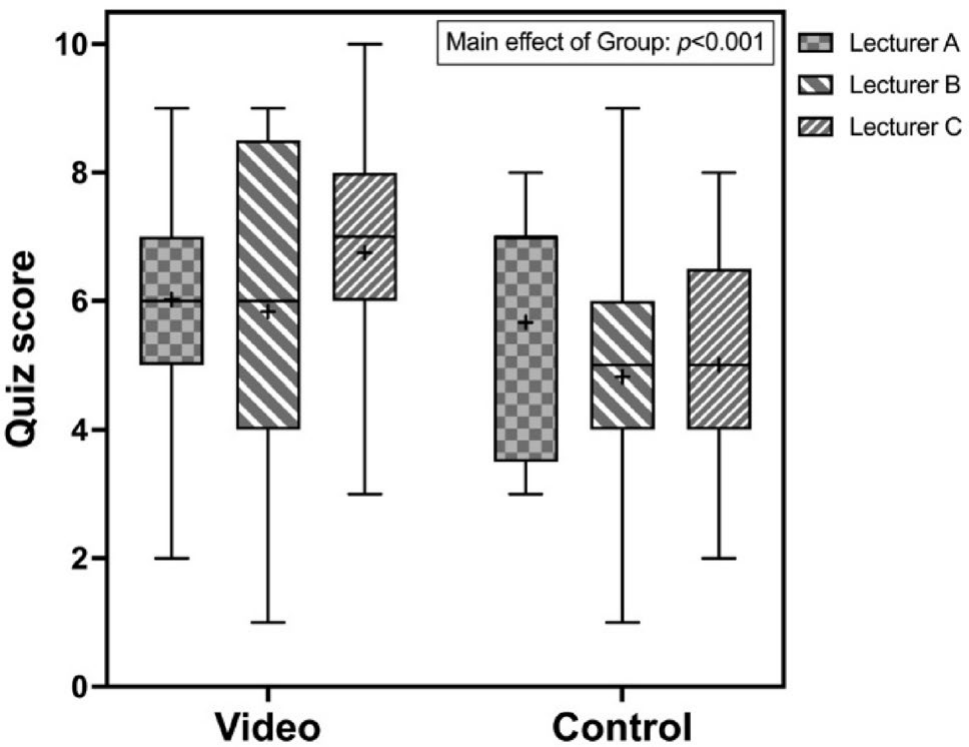
Quiz scores by lecturer and video presence

**Fig. 3:**
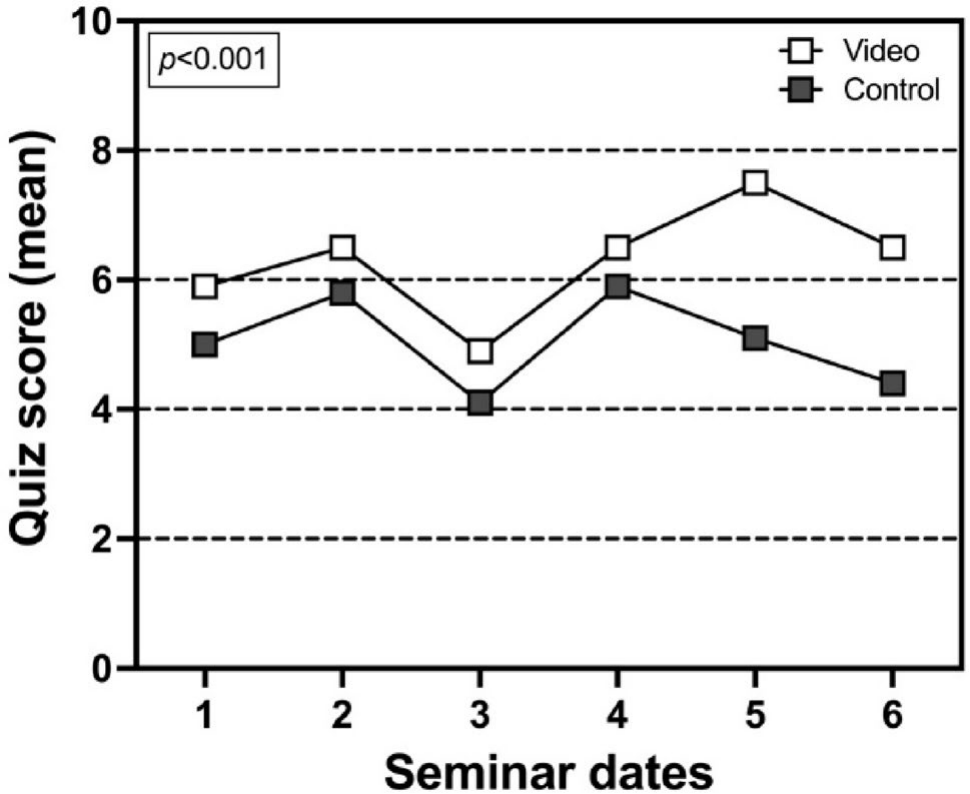
Mean quiz scores across seminar dates by video presence

A two-way ANOVA by group and instructor showed a significant main effect of group (*F*(1, 150) = 16.99, *p* < 0.001), with no significant lecturer effect (*F*(2, 150) = 0.42, *p* = 0.517; Fig. 2). A second ANOVA by group and date showed a significant group effect (*F*(1, 147) = 17.07, *p* = 0.001) and no significant date effect (*F*(5, 147) = 0.01, *p* = 0.919), with no significant interaction (Fig. 3).

For each instructor’s sessions, box plots show the quiz score distribution for the Video and Control Groups. Each box represents the interquartile range (25th–75th percentile); horizontal lines inside boxes indicate the median, and plus signs (+) denote the mean. A two-way ANOVA confirmed a significant main effect of group (*F*(1, 150) = 16.99, *p* < 0.001), with no significant effect of lecturer (*F*(2, 150) = 0.42, *p* = 0.517), indicating that the video-associated difference was consistent across instructors.

Mean quiz scores are shown for students in the Video and Control Groups on each of the six seminar dates. Minor variations in overall score level were observed across dates, but on every date the Video Group outperformed the Control Group. A two-way ANOVA showed a significant main effect of group (*F*(1, 147) = 17.07, *p* = 0.001) and no significant main effect of date (*F*(5, 147) = 0.01, *p* = 0.919), indicating that the video-associated difference was consistent across dates.

### Emotional engagement increased, satisfaction was unchanged

**Fig. 4:**
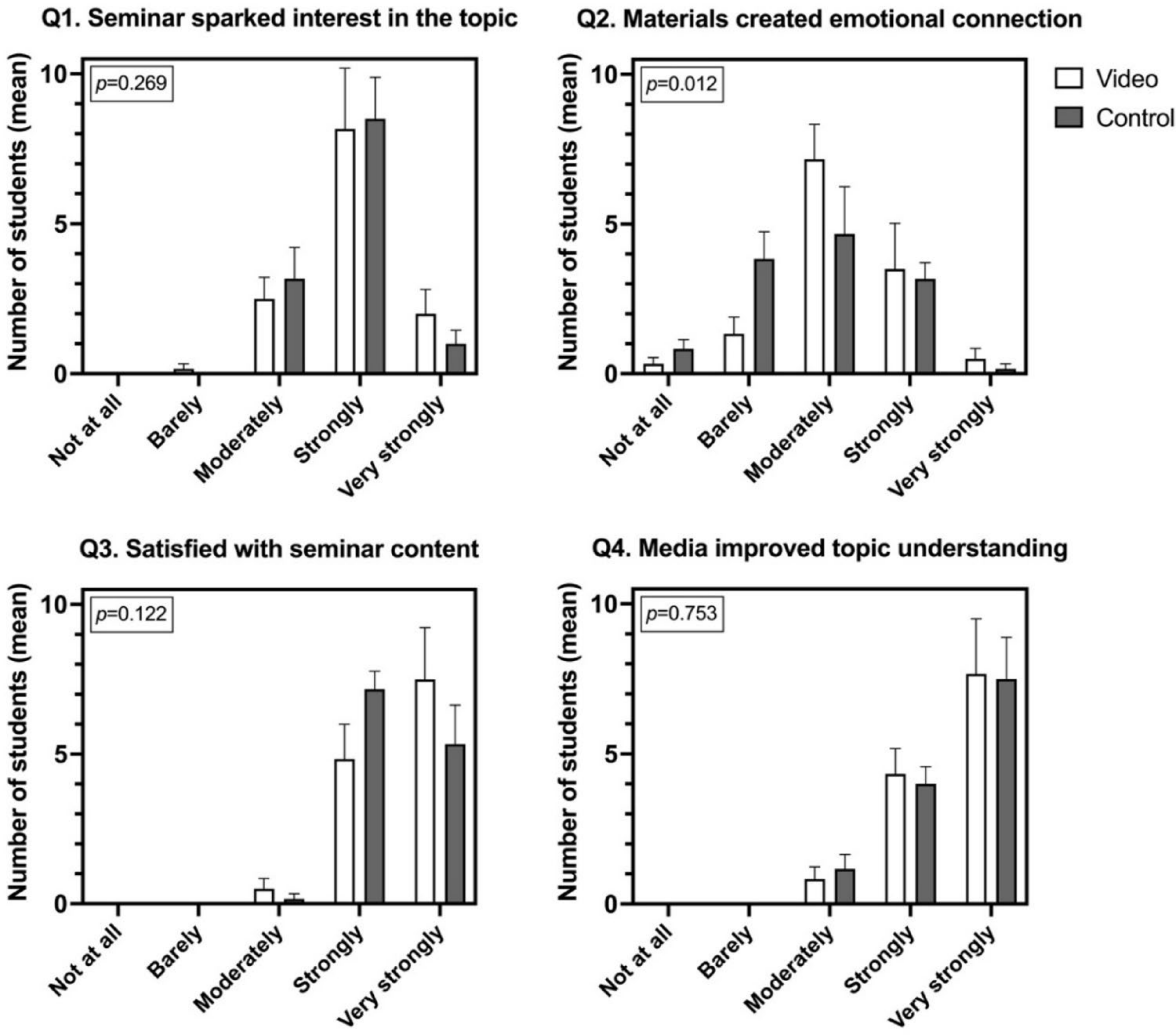
Student evaluations of the seminar experience with and without patient-perspective video

Overall enjoyment was highly positive in both groups. On the yes/no enjoyment item, 100% of students in the Video Group and 99% in the Control Group responded “yes.” On the 5-point Likert-scale items, emotional engagement ratings were higher in the Video Group (*p* = 0.012; Fig. 4), driven mainly by a shift toward “moderate” engagement. No significant differences were found for interest in the topic (*p* = 0.269), overall satisfaction with the seminar (*p* = 0.122), or perceived contribution of media use to understanding (*p* = 0.753; Fig. 4).

Bar graphs display the mean number of students per seminar group selecting each response on a 5-point Likert scale ranging from “not at all” to “very strongly” for four evaluation items: **Q1**, *“The seminar sparked my interest in the topic”*; **Q2**, *“The seminar materials created an emotional connection”*; **Q3**, *“I was satisfied with the seminar content”*; and **Q4**, *“The media used improved my understanding of the topic.”* White bars represent the Video Group (*n* = 77), and gray bars represent the Control Group (*n* = 76). Error bars indicate the standard error of the mean. A Cochran-Armitage trend test showed that students in the Video Group reported significantly stronger emotional connection to the seminar content (Q2, *p* = 0.012). Other items did not differ significantly.

## Discussion

This proof-of-concept study shows that a 4-minute patient-perspective video at the start of a kidney transplantation seminar was associated with higher short-term quiz performance (mean difference 1.2/10; Cohen’s *d* = 0.67) and greater self-reported engagement. Given the brevity, low cost, and scalability of the intervention, a difference of approximately one additional correct answer on a 10-item quiz is educationally meaningful. A plausible mechanism is that the narrative humanized an abstract topic, focused attention, and supported deeper encoding of the seminar content.

These results align with prior work in which patient stories, delivered in person or via video, improved understanding, engagement, and empathy among trainees [11, 12], and in some contexts knowledge [13].

Notably, our video added little factual content and instead presented a patient story in a human-centered manner, yet performance improved. This pattern is consistent with evidence that moderate, personally relevant emotional arousal can enhance attention and memory [8, 14] and with multimedia learning theories, which emphasize the role of affect, attention, coherence, signaling, and active learner processing in learning with media [15]. Importantly, emotional arousal likely follows a curvilinear relation with performance, such that too little or too much may impair learning, suggesting that brief, focused narratives that connect personally relevant goals to upcoming content may be optimal [16].

Higher quiz scores did not translate into higher satisfaction or perceived understanding, echoing prior reports of a disconnect between subjective impressions and objective learning [13]. Ceiling effects and metacognitive miscalibration may contribute to this finding. Our Likert-scale analyses were exploratory, as no adjustment for multiple comparisons was applied. The effect’s consistency across instructors and seminar dates strengthens the interpretation that the video introduction contributed to learning, although causal inference remains cautious given the absence of a pre-test and unlinked quiz/evaluation data.

From a teaching-design perspective, patient-perspective videos can add emotional and contextual depth that complements face-to-face instruction, supporting a blended model [7]. Effects may be strongest for topics with high affective salience, ethical or psychosocial complexity, or limited prior exposure; they may be weaker for procedural topics with low narrative content or among advanced learners who already possess rich schemas, consistent with research on emotion, attention, and memory as well as the expertise reversal effect [17].

Practically, short videos placed at the start of a seminar can prime attention and provide a shared frame, followed by a brief debrief that links the narrative to explicit learning objectives. Avoiding dense factual load within the video may help prevent cognitive overload [15, 18].

To minimize the burden of the study in a compulsory teaching session, we used a single-item engagement measure and a brief objective quiz with predefined learning objectives. This pragmatic approach mirrors routine course evaluation and knowledge testing but does not replace validated instruments or psychometric testing. Immediate post-seminar testing may also favor transient arousal or attention effects over durable learning.

Several limitations should be acknowledged. The single-institution, single-topic design limits generalizability, and only immediate knowledge was measured. Without a pre-test and unlinked quiz/evaluation data, baseline equivalence of the groups and individual-level associations cannot be established. Because clustering at the session level was not modeled, standard errors may be underestimated if intra-cluster correlation was non-trivial, although consistency across instructors and dates is reassuring. The professionally produced video may not reflect effects of lower-production materials. Emotional engagement was measured with a single, non-validated item, and the quiz lacked formal psychometric validation, limiting sensitivity and construct coverage.

Future work should employ validated affect measures and test whether emotional engagement mediates learning effects, for example using the Self-Assessment Manikin, the Positive and Negative Affect Schedule, automated facial coding, or physiological indices [19–21]. Future studies should also include pre-post knowledge assessments with item analysis; examine durability and transfer to attitudes, skills, or career interests; and experimentally vary narrative features such as tone, length, timing, and production quality to identify features that influence learning outcomes.

## Conclusion

A short patient-perspective video at the beginning of a seminar was associated with improved immediate quiz performance and greater self-reported emotional engagement. As a proof-of-concept study, these findings should be replicated with validated affect and knowledge measures, pre-post designs, and longer-term outcomes before broad curricular recommendations are made. Nonetheless, given their low cost and scalability, short patient-perspective videos are a promising adjunct to traditional teaching.

## Supplementary Information

Below is the link to the electronic supplementary material.

Supplementary Material 1. Student evaluation questionnaire for the kidney transplantation seminar (original German version and English translation). The anonymous evaluation form was administered at the end of each seminar to assess students’ subjective impressions of the session. It included five items: one binary question (yes/no) on overall enjoyment of the seminar, and four items using 5-point Likert scales to assess topic interest, emotional connection, satisfaction with content, and perceived contribution of media to topic comprehension. The original questionnaire was in German, with an English translation provided for reference.

Supplementary Material 2. Seminar quiz on kidney transplantation (original German version and English translation). The 10-item multiple-choice quiz was administered at the end of each seminar to assess students’ short-term knowledge retention on the topic of kidney transplantation. Items covered factual content including surgical technique, immunosuppression, complications, legal frameworks, and historical milestones. The quiz was originally in German. Correct answers were indicated on an answer key following the last question.

Supplementary Material 3. Overview of student quiz performance by lecturer and video presence. Summary of the number of students, number of seminar groups, mean number of students per group, mean quiz scores, and standard deviations across all seminars. Data are presented for all students combined and stratified by individual lecturer and by video presence (Video vs. Control)

Supplementary Material 4. Introductory patient-perspective video used in the intervention group. This 4-minute video was shown at the beginning of the seminar to students in the Video Group. It features interviews with a kidney transplant recipient and her mother as the living donor, who describe their personal experience with chronic kidney failure and the impact of transplantation. The narrative is complemented by commentary from the treating surgeon (J.H.) and a voiceover narration by the broadcaster. The original video was presented in German without subtitles.

## Data Availability

No datasets were generated or analysed during the current study.
