# Supplementary material for: Positive impact of a patient-perspective video on engagement and learning in a kidney transplant seminar: a cluster-randomized study
Source: World J Urol. 2026 Aug 17;44(1):577. doi: 10.1007/s00345-026-06680-y (PMC13481544; doi:10.1007/s00345-026-06680-y)
Supplement: Supplementary file 1 [file 345_2026_6680_MOESM1_ESM.docx]

**Questions on Kidney Transplantation**

*Anonymous completion – not graded*

**1. What is the current legal approach to organ donation in Germany?**

A. Opt-out system

B. Limited consent solution

C. Emergency solution

D. Opt-out system with veto rights for relatives

E. Decision-based solution

**2. In which country was the first successful kidney transplantation performed?**

A. France

B. Germany

C. United Kingdom

D. United States of America

E. Canada

**3. Rank the transplant centers by frequency of post-mortem kidney transplants per year (as of 2023), starting with the most frequent from left to right.**

A. Hanover, Berlin, Cologne, Essen, Heidelberg

B. Berlin, Hanover, Heidelberg, Cologne, Essen

C. Hanover, Berlin, Essen, Heidelberg, Cologne

D. Essen, Berlin, Heidelberg, Cologne, Hanover

E. Cologne, Hanover, Berlin, Essen, Heidelberg

**4. Which of the following is the oldest immunosuppressant available after corticosteroids?**

A. Tacrolimus

B. Daclizumab

C. Azathioprine

D. Cyclosporine A

E. Basiliximab

**5. What is tested during the HLA crossmatch before a planned kidney transplantation?**

A. Recipient’s plasma against donor’s serum

B. Recipient’s T-lymphocytes against donor’s plasma

C. Recipient’s platelets with donor’s T-lymphocytes

D. Recipient’s plasma against their own plasma

E. Recipient’s serum against donor’s B- and T-lymphocytes

**6. Which of the following statements about the surgical technique of kidney transplantation is correct?**

A. First the anterior and then the posterior wall of the venous anastomosis is closed.

B. The shorter the artery, the more likely a kinking stenosis is to occur.

C. An end-to-end anastomosis is made between the renal artery and the common iliac artery.

D. A ureteral stricture is more clinically relevant than a ureterovesical reflux.

E. The ureteral anastomosis is sutured using interrupted stitches.

**7. What is the graft survival rate after 5 years?**

A. 90%

B. 80%

C. 70%

D. 60%

E. 50%

**8. Which of the following is not a typical complication after kidney transplantation?**

A. Lymphocele

B. Ureteral implantation stricture

C. Wound healing disorders

D. Macrohematuria

E. Tumor cell dissemination

**9. Which of the following is not a therapeutic option for treating a ureteral stricture?**

A. Psoas hitch surgery

B. Boari flap surgery

C. Nesbit procedure

D. Pyelocystostomy

E. Creation of an ileal interposition

**10. How many countries are currently members of the Eurotransplant International Foundation (Eurotransplant)?**

A. 5

B. 6

C. 7

D. 8

E. 9

*(Correct answers: 1E, 2D, 3B, 4C, 5E, 6D, 7D, 8E, 9C, 10D)*

**Fragen zur Nierentransplantation**

*Anonyme Beantwortung – keine Benotung*

**1. Welche Lösung zur Organspende gilt in Deutschland?**

A. Widerspruchslösung

B. Begrenzte Zustimmungslösung

C. Notstandslösung

D. Widerspruchslösung mit Einspruchsrecht der Angehörigen

E. Entscheidungslösung

**2. In welchem Land erfolgte die erste erfolgreiche Nierentransplantation?**

A. Frankreich

B. Deutschland

C. Großbritannien

D. Vereinigte Staaten von Amerika

E. Kanada

**3. Sortieren Sie die Transplantationszentren nach Häufigkeit ihrer durchgeführten postmortalen Nierentransplantationen pro Jahr (Stand 2023) – beginnend mit dem häufigsten von links nach rechts:**

A. Hannover, Berlin, Köln, Essen, Heidelberg

B. Berlin, Hannover, Heidelberg, Köln, Essen

C. Hannover, Berlin, Essen, Heidelberg, Köln

D. Essen, Berlin, Heidelberg, Köln, Hannover

E. Köln, Hannover, Berlin, Essen, Heidelberg

**4. Welches ist nach den Kortikosteroiden das älteste zur Verfügung stehende Immunsuppressivum?**

A. Tacrolimus

B. Daclizumab

C. Azathioprin

D. Ciclosporin A

E. Basiliximab

**5. Was wird beim HLA-Crossmatch vor einer geplanten Nierentransplantation untersucht?**

A. Blutplasma des Empfängers gegen Blutserum des Spenders

B. T-Lymphozyten des Empfängers gegen Blutplasma des Spenders

C. Thrombozyten des Empfängers mit T-Lymphozyten des Spenders

D. Blutplasma des Empfängers gegen eigenes Blutplasma

E. Blutserum des Empfängers gegen B- und T-Lymphozyten des Spenders

**6. Welche der folgenden Aussagen zur OP-Technik der Nierentransplantation trifft zu?**

A. Es wird erst die Vorderwand und dann die Hinterwand der Venenanastomose verschlossen.

B. Je kürzer die Arterie, desto eher tritt eine Knick-Stenose auf.

C. Es wird eine End-zu-End-Anastomose zwischen Nierenarterie und Arteria iliaca communis hergestellt.

D. Die Harnleiterstriktur ist klinisch relevanter als ein ureterovesikaler Reflux.

E. Die Harnleiteranastomose wird mit Einzelknopfnähten genäht.

**7. Wie hoch ist das Transplantatüberleben nach 5 Jahren?**

A. 90 %

B. 80 %

C. 70 %

D. 60 %

E. 50 %

**8. Welche der folgenden Komplikationen ist keine typische Komplikation nach Nierentransplantation?**

A. Lymphozele

B. Ureterimplantationsstenose

C. Wundheilungsstörungen

D. Makrohämaturie

E. Tumorzellverschleppung

**9. Welche der folgenden Optionen ist keine therapeutische Möglichkeit zur Versorgung einer Harnleiterstriktur?**

A. Psoas-Hitch-Operation

B. Boari-Flap-Operation

C. Operation nach Nesbit

D. Pyelozystotomie

E. Anlage eines Ileuminterponates

**10. Wie viele Länder sind aktuell in der Eurotransplant International Foundation (Eurotransplant) organisiert?**

A. 5

B. 6

C. 7

D. 8

E. 9

*Korrekte Antworten: (1E, 2D, 3B, 4C, 5E, 6D, 7D, 8E, 9C, 10D)*
