# Supplementary material for: Positive impact of a patient-perspective video on engagement and learning in a kidney transplant seminar: a cluster-randomized study
Source: World J Urol. 2026 Aug 17;44(1):577. doi: 10.1007/s00345-026-06680-y (PMC13481544; doi:10.1007/s00345-026-06680-y)
Supplement: Supplementary file 2 [file 345_2026_6680_MOESM2_ESM.docx]

Anonymous seminar evaluation (English translation)

**Did you enjoy the seminar on kidney transplantation?**

☐ No     ☐ Yes

**To what extent did the seminar spark your interest in the topic of kidney transplantation?**

☐ Not at all  ☐ Barely  ☐ Moderately  ☐ Strongly  ☐ Very strongly

**To what extent did the teaching materials create a personal or emotional connection to the topic of kidney transplantation?**

☐ Not at all  ☐ Barely  ☐ Moderately  ☐ Strongly  ☐ Very strongly

**How satisfied were you with the content of the seminar?**

☐ Not at all  ☐ Barely  ☐ Moderately  ☐ Strongly  ☐ Very strongly

**To what extent did the use of media (e.g., videos) improve the comprehensibility of the topic?**

☐ Not at all  ☐ Barely  ☐ Somewhat  ☐ Strongly  ☐ Very strongly

Anonyme Bewertung des Seminars (German original version)

**Hat Ihnen das Seminar zur Nierentransplantation gefallen?**

☐ Nein     ☐ Ja

**Wie sehr hat das Seminar Ihr Interesse an dem Thema Nierentransplantation angeregt?**

☐ Überhaupt nicht  ☐ Kaum  ☐ Mäßig  ☐ Stark  ☐ Sehr stark

**Wie sehr ist bei Ihnen ein persönlicher bzw. emotionaler Bezug zu dem Thema Nierentransplantation durch die verwendeten Lehrmaterialien entstanden?**

☐ Überhaupt nicht  ☐ Kaum  ☐ Mäßig  ☐ Stark  ☐ Sehr stark

**Wie stark haben die Lehrinhalte des Seminars zu Ihrer Zufriedenheit beigetragen?**

☐ Überhaupt nicht  ☐ Kaum  ☐ Mäßig  ☐ Stark  ☐ Sehr stark

**Wie sehr hat die Verwendung von Medien (z. B. Videos) die Verständlichkeit des Themas verbessert?**

☐ Überhaupt nicht  ☐ Kaum  ☐ Etwas  ☐ Stark  ☐ Sehr stark
