# Supplementary material for: Positive impact of a patient-perspective video on engagement and learning in a kidney transplant seminar: a cluster-randomized study
Source: World J Urol. 2026 Aug 17;44(1):577. doi: 10.1007/s00345-026-06680-y (PMC13481544; doi:10.1007/s00345-026-06680-y)
Supplement: Supplementary file 3 [file 345_2026_6680_MOESM3_ESM.docx]

|  | | Students | Groups | Students per group (mean) | Point score (mean) | Point score (SD) |
| --- | --- | --- | --- | --- | --- | --- |
| Both groups | lecturers combined | 154 | 12 | 12.8 | 5.55 | 1.88 |
|  | lecturer A | 56 | 4 | 14 | 5.98 | 1.61 |
|  | lecturer B | 69 | 5 | 13.8 | 5.01 | 1.97 |
|  | lecturer C | 29 | 3 | 9.7 | 5.97 | 1.88 |
| Video | lecturers combined | 77 | 6 | 12.8 | 6.14 | 1.79 |
|  | lecturer A | 48 | 3 | 16 | 6.00 | 1.57 |
|  | lecturer B | 13 | 1 | 13 | 5.92 | 2.47 |
|  | lecturer C | 16 | 2 | 8 | 6.75 | 1.73 |
| Control  (No video) | lecturers combined | 77 | 6 | 12.8 | 4.95 | 1.78 |
|  | lecturer A | 8 | 1 | 8 | 5.88 | 1.96 |
|  | lecturer B | 56 | 4 | 14.3 | 4.80 | 1.79 |
|  | lecturer C | 13 | 1 | 13 | 5.00 | 1.63 |

**Supplementary Table S1.**
